# New Remedies

**Published:** 1886-05

**Authors:** C. J. Boyd Wallis


ARTICLE IV.

### NEW REMEDIES.

BY C. J. BOYD WALLIS, L. D. S., ENG., &C.

Mr. T. Christy, F. L. S., of the enterprising firm of Christy & Co., Drug Importers, Fenchurch Street, and author of "New Commercial Plants and Drugs," and others of the drug trade, are continually introducing new medicinal drugs and chemicals to the notice of the profession, and it is well for us to take note of them, that we may glean from them those of special value as additions to our Dental *Materia Medica*, for some of these new remedies might be employed with advantage to a much greater extent than they are in dental surgery.

ACETATE OF ALUMINIUM is a valuable antiseptic and deodorant, that has been too much over-looked by the medical and dental professions, and this neglect is greatly due to the difficulty of preparing, at a low price, a perfectly neutral solution, and also to the fact that its value as a deodorizer of blood, used in the purification of sugar, was long kept a secret in the interests of sugar refiners. The value of the acetate for purposes of embalming was made known by Gammal in 1827; Burow, in 1857, called attention to its value for destroying the disagreeable odor of gangrenous wounds, and Professor Bruns confirms the statement, while Professor Bilbroth asserts its value as an antiseptic to be equal to that of carbolic acid. Dr. Brun states that during the twenty years in which he had treated wounds with the acetate, he had not seen a single death from pyæmia, even under the most disadvantageous conditions. Dr. A. Rose, of New York, strongly recommends it as an antiseptic and deodorant, and gives the following method of preparing it for medical purposes:—Ten parts of sulphate of aluminium are dissolved in the least possible quantity of hot water, and 17 parts of crystallized acetate of lead are added, also dissolved in hot water. The two solutions are then mixed. The sulphate of lead is allowed to settle, and the decanted supernatant liquid is treated with hydrogen sulphide, and after filtration, to remove the lead, it is warmed until the odor of the gas is driven off. The liquid is then diluted until it measures 48 parts. One ounce of the liquid should then contain a drachm of anhydrous acetate of aluminium. Thus prepared, it has a specific gravity of 1.0392. It can be obtained in the form of scales, soluble in water, in the same way as other scaled preparations.

Dr. Wilson, of Aix-la-Chapelle, employs acetate of aluminium in solution as a mouth wash during the administration of mercury in syphilitic diseases, and states that by its use salivation is prevented. He uses the following formulæ:

| | |
|---|---|
| Acetate of Lead.............................. | 240 grs. |
| Powdered Alum............................. | 330 " |
| Distilled Water.............................. | 16 ozs. |
| Aromatic or Peppermint Water........... | 8 " |

Dissolve the lead and alum salts separately in the water; mix and stir well together; either filter or allow the precipitate to settle, and decant the clear solution, to which add the aromatic or peppermint water. It may be used in a more diluted form if necessary, and the mouth should be regularly rinsed out from the beginning of the treatment to the end a dozen times a day, or even more, and in urgent cases during the night; if by any means salivation should occur, its ill effects are counterbalanced by the use of the aluminium wash. A 2 per cent. solution is said to be sufficient to permanently protect organic substances from putrefaction, and for purposes of irrigation a 1 or ½ per cent. solution may be used.

I have prescribed the following formulæ, which I have found to be a most excellent mouth wash, and at the same time a useful preservative of anatomical specimens and other organic substances:—

| | |
|---|---|
| Acetate of Aluminium | 32 grs. |
| Boric Acid | 64 " |
| Glycerine, pure | 1 oz. |
| Oil of Eucalyptol | 10 m |
| Engenol | 6 " |
| Eau de Cologne | 1 oz. |
| Chloroform Water | to 6 " |

Mix. This may be used as it is or diluted, as required.

The next preparation to notice is IODOFORM: tri-iodomethane, $C H I_3$.—This is not, strictly speaking, a new preparation, but a new process has been introduced by which an absolute iodoform—that is an absolutely pure product—is obtained by means of electrolysis. A sample has been supplied to me by Messrs. Zimmermann, agents in London for the manufacturers, Schering & Co., of Berlin. I regret that I have been unable to ascertain the exact method of the electrolytic process employed in its production. It is possibly a trade secret. But it is probably obtained by precipitation, by passing a constant current of electricity through a watery-alcoholic solution of iodide of potassium to which a uniform supply of carbonic acid is admitted; at any rate, chloroform, iodoform, and bromoform may be obtained by passing an electric current through a hot strong alcoholic solution of chloride, iodide, or bromide of potassium respectively, carbonic anhydride being simultaneously supplied.

In reference to the deodorization of iodoform, so many things have been suggested for this purpose—notably, otto of rose, sanitas oil, tannic acid, Tonquin bean, balsam of Peru, and carbolic acid—but all of these are more or less failures; the most successful in my hands being carbolic acid, yet even this takes some few weeks to act before deodorization is complete. Dr. Putz, of Graefrath, says that he confines himself to oil of mirbane or nitrobenzol for this purpose, all other deodorants having failed in his hands. He uses six drops of nitrobenzol for every gramme of iodoform.

Coffee, a more perfect deodorizer than any of the foregoing, has been suggested by Dr. Oppler, and he has found that from 20 to 50 per cent. effectual for this purpose. It is said that coffee. when roasted, is an excellent application for wounds, and the effect is attributed to the presence of vegetable charcoal and to the aromatic empyreumatic compounds formed during the roasting process. A compound of 50 parts of iodoform and 25 parts of finely powdered coffee, triturated with a few drops of ether and then dried, will be found effective. A useful external application may be made by combining

| | |
|---|---|
| Iodoform | 1.00 grms. |
| Paraffin | 10.00 " |
| Powdered Roasted Coffee | 0.30 " |

Mix.

The taste of castor oil may be disguised by combining

| | |
|---|---|
| Castor Oil | 3 parts. |
| Powdered Roasted Coffee | 1 " |
| Powdered Sugar | to taste. |

Mix.

Other drugs of an unpleasant taste or odor may be combined in a similar manner.

IODOL: Tetraiodpyrrol, $C_4I_4NH$.—Another antiseptic has been more recently introduced and favorably reported upon by Dr. Mazzoni, of Rome; it is, I think, a preparation which we shall find of the greatest value in the practice of our specialty, for it has this great advantage over iodoform, that it is free from the unpleasant and penetrating odor of the latter, and it does not produce any symptoms of intoxication, while it contains about 90 per cent. of iodine, only 7 less than iodoform.

*Iodol* is a tetraiodpyrrol. Pyrrol is one of the constituents of animal oil, the distillate obtained by subjecting animal substances containing protein bodies to destructive distillation. When this is freed from impurities and then precipitated by iodide of potassium, an iodine substitution product is obtained, namely, tetra-iodpyrrol, and this product has been called *Iodol*, for brevity's sake, by the discoverers, Drs. Silber and Ciamician, of Rome. Messrs. Kalle & Co., of Biebrich on the Rhine, are now manufacturing it on a large scale, and I suspect it will soon be as readily obtained through the usual channels as iodoform.

*Iodol* forms a light brownish micro-crystalline powder, free from taste, having a faint odor resembling thymol, and upon heating to a temperature of 100° C. iodine vapor is evolved. It is almost insoluble in water, moderately soluble in hot oil, freely soluble in ether, chloroform, and in three parts of alcohol; more soluble in absolute alcohol. It is not precipitated from a alcoholic solution by the addition of glycerine, but it is by the addition of water. Sulphuric acid dissolves it with a green color, and nitric acid changes a heated alcoholic solution to a bright red. It possesses antiseptie properties similar to iodoform, exercises a local anæsthetic action, and greatly promotes the granulation of wounds.

Naphthol.—*b*-Naphthol: Syn. naphthol alcohol. Naphthol is another important product; but it has been but little used in the dental surgery up to the present time, yet it is, I consider, second to neither of the foregoing in its value as a therapeutic agent in the treatment of the teeth. This preparation I have now employed for a considerable time, and I have found it invaluable as an antiseptic, disinfectant, and deodorant. It is especially as the latter that I have used it, and with the best results.

The preparation I employ is a pure re-sublimed *b*-naphthol. It is a derivative of coal-tar, and is in white shining laminar crystals, having an odor similar to storax. It has a sharp burning taste, and its powder excites violent sneezing. It is very soluble in ether, chloroform, and benzol; slightly soluble in hot water; soluble in an equal weight of alcohol, and in 1 part in 8 of olive oil and lard, and in 1 part in 80 of vaseline. It sublimes on heating, and may be distilled with steam, a property to be remembered when making hot solutions, or loss will occur.

*Naphthols* are compounds derived from naphthalin by the substitution of one molecule of hydroxyl (H. O.) in place of one of hydrogen. There are two naphthols, *a* and *b* (alpha and beta) naphthol, so named for the purposes of distinction. They are respectively formed by fusing the two acids, alpha-naphthalin-sulphonic acid and beta-naphthalin-sulphonic acid, with alkalies, whereby hydroxyl is substituted for the sulphonic acid (H. S. O3), *a*-naphthol is with difficulty soluble in hot water; easily soluble in alcohol and ether. *B*-naphthol, owing to the greater stability of the *b*-naphthalin-sulphonic acid, is more easily prepared, and it is this product that is generally sold in trade as naphthol. When pure, it is white, melts at 122° C., boils at 286° C., and on the addition of ferric-chloride its solution is changed to a greenish color, while *white* di-naphthol is separated; and with the addition of ferric-chloride to an aqueos solution of *a*-naphthol, *violet* flakes of di-naphthol are thrown down.

*Naphthol* being odorless and colorless, gives it some advantages in the treatment of skin diseases, for which it has been successfully employed in advanced scabies, eczema, psoriasis, and for hyperidrosis, chronic ulcerations in ano, urinary fistula, abscesses, necrosis, &c., it has also been used to disinfect rooms, for which it is excellent and specially suited, as it destroys all offensive odors, and not, I think, as some so-called disinfectants do, by overcoming a lesser odor with their stronger odor, but by chemical action: and for this purpose it may be sublimed by means of a spirit lamp placed beneath a dish containing the naphthol; the crystals of naphthol in minute subdivision are thus driven off by the heat, and penetrate every portion of the room, which becomes perfectly deodorized. If applied in solution (15 to 20 grains to a pint,) it encourages healthy granulations in wounds, and stimulates the growth of new tissue.

The following compound is similar, but of a somewhat milder form, to that which I communicated to the *Lancet* some months ago. It is an invaluable, agreeable and refreshing application for fœtid odors of the feet and body arising from excessive perspiration, wounds, &c.

The formulæ is as follows:—

| | |
|---|---|
| Naphthol resublimed | 5 parts. |
| Salicylic acid | 5 " |
| Boric acid | 10 " |
| French chalk, pure | 80 " |
| Engenol | 5 ". |

Mix. Pertume if desired.

A useful formulæ for an external application for eczema, hyperidrosis of the hands, feet, &c., may be composed as follows:—

| | |
|---|---|
| Naphthol resublimed | 5 parts. |
| Glycerine, pure | 11 " |
| Engenol | 4 " |
| Alcohol | 80 " |

Mix.

This brings me to a compound which I have long used in the treatment of the teeth, and for the formula of which I have frequently been asked. It is as follows:—

| | |
|---|---|
| Naphthol resublimed | 3 drachms. |
| Iodol | 1 " |
| Menthol | 2 " |
| Carbolic acid, pure | 4 " |
| Engenol and Eucalyptol aa | 2 " |

Chloroform, 6 drachms; absolute alcohol, ad. 3 ounces. Mix.

For a permanent dressing for nerve canals I employ a similar preparation of a stronger character, made by the addition of another dram each of iodol and naphthol and two drams of gum sandarach.

This preparation, using until lately iodoform instead of iodol, I have found of the utmost value. It is at once a powerful antiseptic, disinfectant, and deodorizer, and an excellent application for a painful tooth. Space will not permit me to enter into an account of the experiments I have made with naphthol preparations upon teeth in the mouth and out of it; but I may mention that I have filled nerve canals of extracted teeth with this preparation, and filled over it in the usual manner, and placed these teeth in various solutions and in human saliva for various periods of from three months to two years, and have then broken these teeth open at the end of the respective periods, and have found the dressing in every case perfectly sweet. I am of opinion that we have in naphthol the most durable and permanent antiseptic of all other preparations for the treatment of the teeth with which I am acquainted, with, perhaps, the one exception of cupric oxide, for it is not decomposed or rendered inert by contact with organic matter or the products of putrefaction.

HYDRONAPHTHOL.—In English and foreign journals attention has been recently called to this preparation which, it is said, occurs in commerce as a grey powder of a micaceous appearance, having a faint odor resembling naphthaline. Dr. Fowler recommends it very strongly as an antiseptic; it is sparingly soluble in water, but dissolves freely in alcohol, ether, chloroform, and fixed oils. It has been described as belonging to the aromatic series, and bearing the same relation to the hypothetical radical naphthyl as carbolic acid does to phenyl. A saturated aqueous solution (viz., 1 in 1,000) is stated to be perfect in its inhibitory action, and to preserve animal tissues and liquids perfectly for an indefinite period, although producing no other perceptible effect upon living tissue than coating it with a slight film. It is said to be non-poisonous, non-irritant, and non-corrosive; having an antiseptic power second only to mercuric chloride, and ten times greater than carbolic acid. It fuses between 113° and 118° C., gives off vapor at 126° C., commences to sublime at 130° C., and sublimes all but a dark carbonaceous residue between 140° and 145° C., the sublimate forming small, colorless rhombic plates. A powder composed of two per cent. of hydronaphthol with carbonate of magnesium or silicious earth is stated to have advantages over iodoform. It has been said that hydronaphthol corresponds with impure *b*-naphthol. I have not tested the value of hydronaphthol, but if these assertions are correct they bear out my statements in reference to the value of *b*-naphthol. —*London Dental Record.*

---

ARTICLE V.

